# Annexin-A1 enhances breast cancer growth and migration by promoting alternative macrophage polarization in the tumour microenvironment

**DOI:** 10.1038/s41598-017-17622-5

**Published:** 2017-12-20

**Authors:** Leonardo A. Moraes, Shreya Kar, Sok Lin Foo, Tong Gu, Yi Qian Toh, Patrick B. Ampomah, Karishma Sachaphibulkij, Gracemary Yap, Olga Zharkova, Hakim M. Lukman, Anna-Marie Fairhurst, Alan Prem Kumar, Lina H. K. Lim

**Affiliations:** 1 Department of Physiology, Yong Loo Lin School of Medicine, National University of Singapore (NUS), Singapore, Singapore; 2 NUS Immunology Program, Life Sciences Institute, NUS, Singapore, Singapore; 3 Cancer Science Institute of Singapore, NUS, Singapore, Singapore; 4 Department of Pharmacology, Yong Loo Lin School of Medicine, NUS, Singapore, Singapore; 5 NUS Graduate School for Integrative Sciences and Engineering, NUS, Singapore, Singapore; 6 Singapore Immunology Network (SIgN), Agency for Science, Technology and Research, Singapore, Singapore; 7 Medical Science Cluster, Yong Loo Lin School of Medicine, National University of Singapore, Singapore, Singapore; 8 Curtin Medical School, Faculty of Health Sciences, Curtin University, Perth, WA Australia

## Abstract

Macrophages are potent immune cells with well-established roles in the response to stress, injury, infection and inflammation. The classically activated macrophages (M1) are induced by lipopolysaccharide (LPS) and express a wide range of pro-inflammatory genes. M2 macrophages are induced by T helper type 2 cytokines such as interleukin-4 (IL4) and express high levels of anti-inflammatory and tissue repair genes. The strong association between macrophages and tumour cells as well as the high incidences of leukocyte infiltration in solid tumours have contributed to the discovery that tumour-associated macrophages (TAMs) are key to tumour progression. Here, we investigated the role of Annexin A1 (ANXA1), a well characterized immunomodulatory protein on macrophage polarization and the interaction between macrophages and breast cancer cells. Our results demonstrate that ANXA1 regulates macrophage polarization and activation. ANXA1 can act dually as an endogenous signalling molecule or as a secreted mediator which acts via its receptor, FPR2, to promote macrophage polarization. Furthermore, ANXA1 deficient mice exhibit reduced tumour growth and enhanced survival *in vivo*, possibly due to increased M1 macrophages within the tumor microenvironment. These results provide new insights into the molecular mechanisms of macrophage polarization with therapeutic potential to suppress breast cancer growth and metastasis.

## Introduction

Macrophages are heterogeneous cells that are released from the bone marrow as immature monocyte and after circulating in the blood vessels, migrate into target tissues to undergo final differentiation into mature macrophages^1,2^. Monocytes and macrophages play a critical role in tissue remodelling, inflammation and immunity including phagocytosis and secretion of cytokines, proteases and growth factors^2,3^. The complex interplay between cancer cells and the host immune response has been well established, supporting the concept that disruption of endogenous mechanisms that promotes resolution of inflammation, could result in tumour progression^4–6^. Therefore, macrophages, as key regulators of host immunity, have a pivotal role on the tumour microenvironment^3^.

The paradoxical interaction between macrophages and cancer reflect the functional plasticity of these cells. Macrophages can differentiate into a spectrum of distinct, functional phenotypes in response to signals present within individual microenvironments, namely the classically activated macrophages (M1), and alternatively activated macrophages (M2). The M1 phenotype has potent microbicidal activity, promotes Th1 responses and are characterized by the production of pro-inflammatory factors such as IL-12 and tumour necrosis factor-α (TNF-α), whereas the M2 phenotype drives angiogenesis, tumour progression and promotes Th2-type adaptive immune responses, secreting IL-10 and transforming growth factor-β (TNF- β)^3,7,8^.

The M2 phenotype can be further subdivided into M2a, M2b, M2c and M2d cells according to different stimuli^9,10^. M2a and M2b macrophages promote immune regulatory functions and drive Th2 response, whereas M2c macrophages have a predominant role in suppressing inflammation and promoting tissue remodelling. M2d macrophages, also termed tumour-associated macrophages (TAMs), are a major source of vascular endothelial growth factors, which accumulate at the tumour site by tumour-derived signals, such as macrophage colony stimulating factor (M-CSF/CSF-1), vascular epithelial growth factor (VEGF) and chemokines CCL2 and CCL5)^3,10,11^. The strong correlation between infiltrated TAMs and tumour cells promote tumour growth and vasculature^11–13^. Furthermore, clinical studies have demonstrated that the presence of TAMs is strongly correlated with poor prognosis in breast cancer patients indicating advanced tumour progression and metastasis^14^, however the underlying signals and mechanism which TAMs provide to the tumour cells to promote metastasis still remains to be established.

Annexin 1 (ANXA1), a highly abundant 37-kDa protein member of the annexin superfamily, was initially shown to be a glucocorticoid-regulated phospholipase A_2_ (PLA_2_) inhibitor^15^. The annexin superfamily of calcium and phospholipid-binding proteins has been implicated in many physiological processes, including differentiation, apoptosis, proliferation and inflammation. In particular, ANXA1 is anti-inflammatory, pro-apoptotic and regulates differentiation^16–20^. We have previously demonstrated that ANXA1 can associate with NF-κB and increase c-Myc activity leading to the inhibition of miR196a transcription, inducing a negative feedback loop to promote breast cancer migration and metastasis^21,22^. ANXA1 is highly expressed in metastatic and triple negative (estrogen, progesterone and HER2 receptor) breast cancer and it has been reported to promote tumour development and progression^23^. The biological effects of ANXA1 are mediated through a family of G-protein-coupled receptors (GPCRs) known as the formyl peptide receptors (FPRs) that includes FPR1, FPR2 (also known as FPR2/ALX or formyl peptide receptor like-1) and FPR3^24^. FPRs were originally identified in phagocytic leukocytes and can mediate cell chemotaxis and activation in response to bacterial peptides such as F-Met-Leu-Phe (fMLP)^25^. In addition to the important roles in inflammation, FPR is also implicated in cancer^26^. FPR1 has been shown to be expressed by highly malignant glioblastoma (GBM) cells and can promote chemotaxis through the activation of the EGF receptor^27^. Indeed, previous reports have demonstrated that ANXA1 and lipoxin-A4 (LXA4) can activate FPR2 to promote breast cancer proliferation^8,28^. In this report, we show an underlying mechanism that demonstrates that macrophage ANXA1 is required for and plays an important role in the tumour microenvironment, and is important in the induction of expression of M2 macrophage subset markers.

## Results

### 4T1 and 67NR murine breast cancer cells polarize macrophages to M2-phenotype

4T1 and 67NR breast cancer cell lines were derived from a single mammary tumour that develop spontaneously in a BALB/c mouse. 4T1 cells are highly invasive leading to metastases, whereas 67NR cells were derived from primary tumours, but do not metastasize or invade^29,30^.

**Figure 1:**
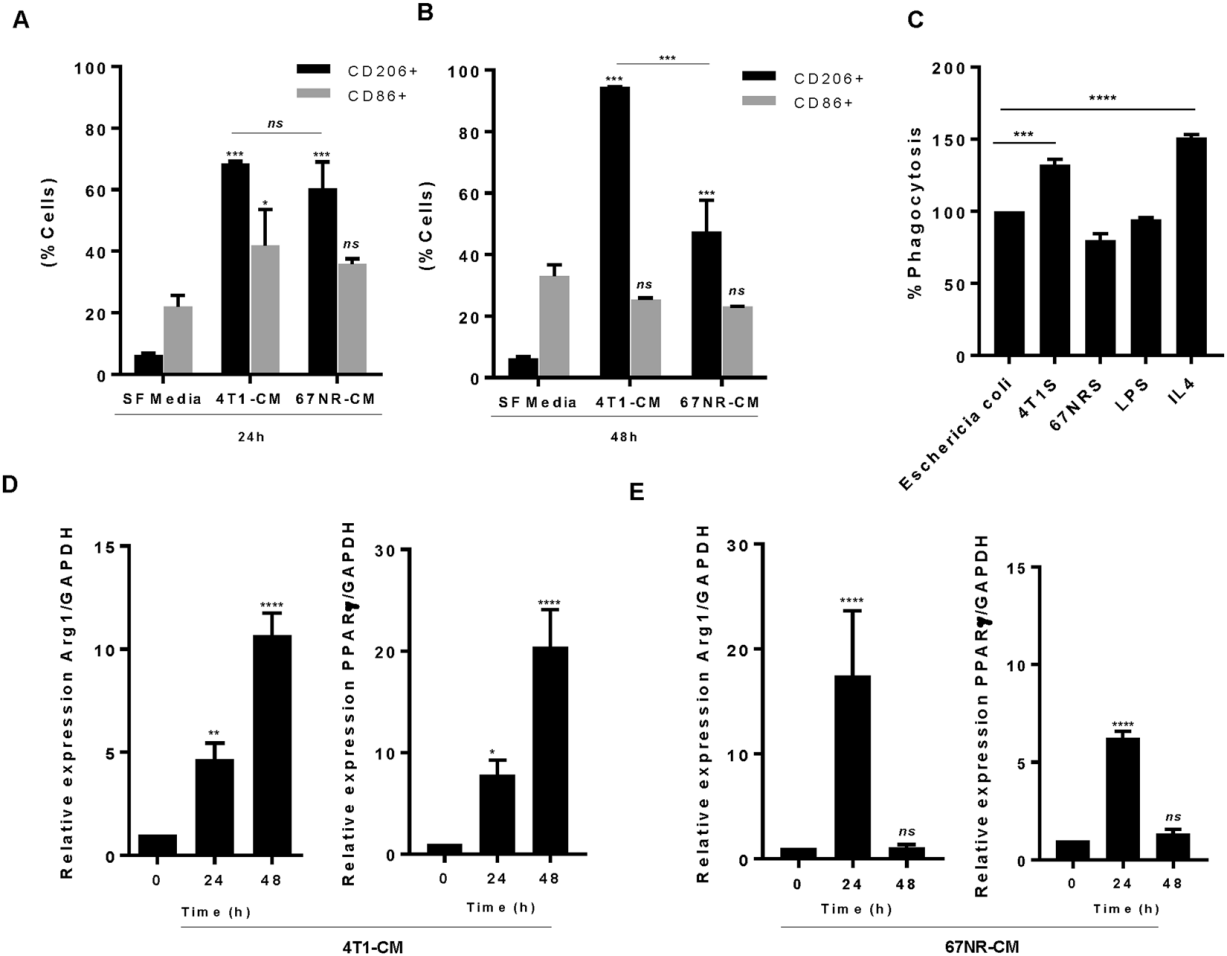
4T1 and 67NR-conditioned media effects on macrophage polarization. Flow cytometry analysis shows expression of CD86 (M1), CD 206 (M2) in macrophages exposed to 4T1 and 67NR-conditioned media or serum-free media (control) for 24 h (**A**) and 48 h (**B**). (**C**) Naïve RAW 264.7 macrophages were treated with 4T1, 67NR,, IL4 (20 ng mL^−1^) or LPS (10 ng mL^−1^) for 24 h prior incubation with *E. coli* bioparticles. *E. coli* phagocytosis was determining by measuring bioparticles fluorescence. (**D**,**E**) Phenotype for distinct macrophages were determined by mRNA expression of Arg1 and PPARγ after treatment with 4T1 and 67NR-conditioned media for 24 h and 48 h. Data represents relative gene expression after normalization with GAPDH by RT-qPCR (ΔΔCt method). Data shown are mean ± SD (n = 4). *P ≤ 0.05, **P ≤ 0.01, ****P ≤ 0.001, ANOVA.

Breast tumours are highly infiltrated by different types of host leukocytes, and importantly, T cells and monocytes which differentiate into TAMs at the tumour site^30^. Given the ability of TAMs to be recruited to tumours by a range of growth factors and chemokines, which are often produced by the tumour cells themselves, we sought to determine the effect of 4T1 and 67NR-conditioned media (CM) on macrophage polarization. The proportion of M1 and M2 macrophages expressing specific M1 (CD86) or M2 (CD206) cell surface markers was assessed by FACS analysis (Fig. 1A,B).

Raw264.7 macrophages treated with 4T1-CM exhibited a significant increase of CD206 expression compared to control (68.5% ± 0.6 vs 6.3 ± 0.6 at 24 h; 99.4 ± 0.1 vs 6.2 ± 0.7 at 48 h). CD206 expression decreases upon 67NR-CM treatment as compared to 4T1-CM (47.5 ± 10 vs 99.4 ± 0.1 at 48 h). Macrophages treated with 67NR-CM did not exhibit any difference in CD86 expression compared to control (35.8 ± 1.6 vs 22 ± 3.7 at 24 h; 23.1 ± 0.1 vs 33 ± 3.6 at 48 h). In addition, CD86 expression was reduced after 4T1-CM treatment, suggesting that after 24 h, macrophages exhibited a polarization shift with an increase of CD206 expression and reduction in CD86 expression.

Next, we examined if 4T1-CM could enhance the phagocytic function of macrophages. RAW 264.7 mouse macrophages were treated with 4T1, 67NR-conditioned culture media, LPS (10 ng mL^−1^) or IL4 (20 ng mL^−1^) for 24 h prior to incubation with *E. coli* bioparticles (Fig. 1C). Treatment of RAW cells with 4T1-CM enhanced the phagocytic activity by 30% compared to the control. This effect was similar in macrophages treated with IL4 for 24 h, our positive control for alternatively activated, or M2 macrophages. However, treatment of macrophages with 67NR-CM or LPS did not enhance phagocytic ability, suggesting that alternatively activated macrophages, through the activation with 4T1-CM or IL4 exhibit higher phagocytic ability.

The extent of macrophage polarization by 4T1 and 67NR-CM was also confirmed at the gene expression level. As expected, M2 polarization was observed in RAW macrophages following treatment with IL4 (Supplemental data Fig. 1A,B). A time-dependent increase in the expression of M2-signature mRNAs (Arginase-1 and PPARγ) was observed with 4T1-CM treatment, with the highest expression at 48 h post-treatment. (Fig. 1D). In comparison, arginase-1 and PPARγ expression was significantly increased 24 h post-treatment with 67NR-CM, which was shortlived and reduced back to control levels by 48 h (Fig. 1E). M2 polarization status at protein level after 4T1-CM treatment was confirmed by examining the expression of Arg1, PPARγ and iNOS by immunoblotting analysis (Supplemental data Fig. 1C). Consistent with mRNA expression, stimulation with 4T1-CM for 48 h induced high expression of M2 markers compared to control, while 67NR-CM treatment did not change iNOS, Arg-1 or PPARγ expression at this time point. These results demonstrate that treatment of macrophages with breast tumor conditioned media, in particular 4T1, can induce a skewing to the M2 subset with different kinetic profiles.

### M2 polarization is positively correlated with tumour growth in a MMTV-Wnt mouse model

**Figure 2:**
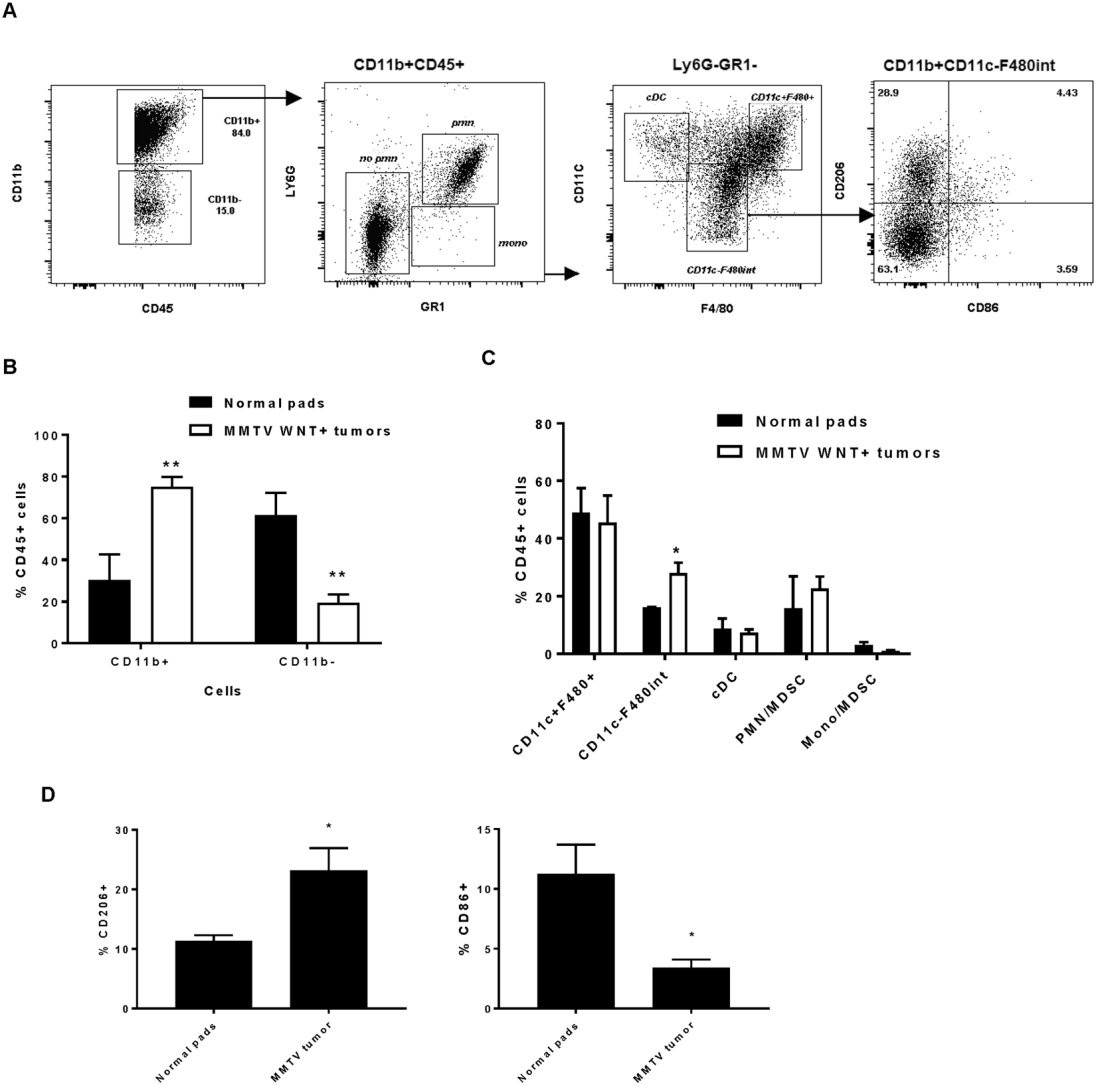
Populations and phenotypes of macrophages derived from MMTV-WNT + breast tumours. (**A**) Representative flow cytometry plots showing gating of CD11b+CD45+ cells in MMTV-WNT+ tumours. (**B**) Analysis of the percentage of CD45+ cells which are CD11b+ or CD11b-. (**C**) Different populations of CD11b+ CD45+ cells. (**D**) Percentage of CD206+ (M2) or CD86+ (M1) macrophages obtained from CD11c-F480int macrophages from breast tumours on MMTV mice or mammary fat pads of B6 control female mice. Data shown are mean ± SD (n = 4). *P ≤ 0.05, **P ≤ 0.01, ANOVA.

A spontaneous mammary tumour model – MMTV-Wnt has been used previously to examine the genetic basis of breast cancer. It was reported that transgenic expression of Wnt using MMTV LTR enhances ductal hyperplasia in early life and 50% of female transgenic mice exhibit mammary adenocarcinomas by 6 months^31^. We next determined the macrophage immune signature of the primary tumour in MMTV-Wnt mice by analysis of cell numbers and phenotype. Phenotypic analysis of TAM derived from MMTV-Wnt mice was performed on isolated macrophages for distinct populations using surface expression markers by flow cytometry. We found that there were more CD11b- leukocytes in normal mammary glands while more leukocytes in MMTV-WNT mammary tumours were CD11b+ (Fig. 2B). In the CD11b^+^ enriched population, there were significantly more CD11b+ Ly6G-GR1-F480intCD11c- macrophages in tumours compared to normal mammary glands (Fig. 2C), which may be recruited macrophages and more of these CD11c-F480int macrophages exhibit high expression of CD206 and low expression of CD86 (Fig. 2A,D), suggesting that TAMs in the MMTV-Wnt tumours may be more M2 polarized.

### ANXA1 plays a role in macrophage polarization

**Figure 3:**
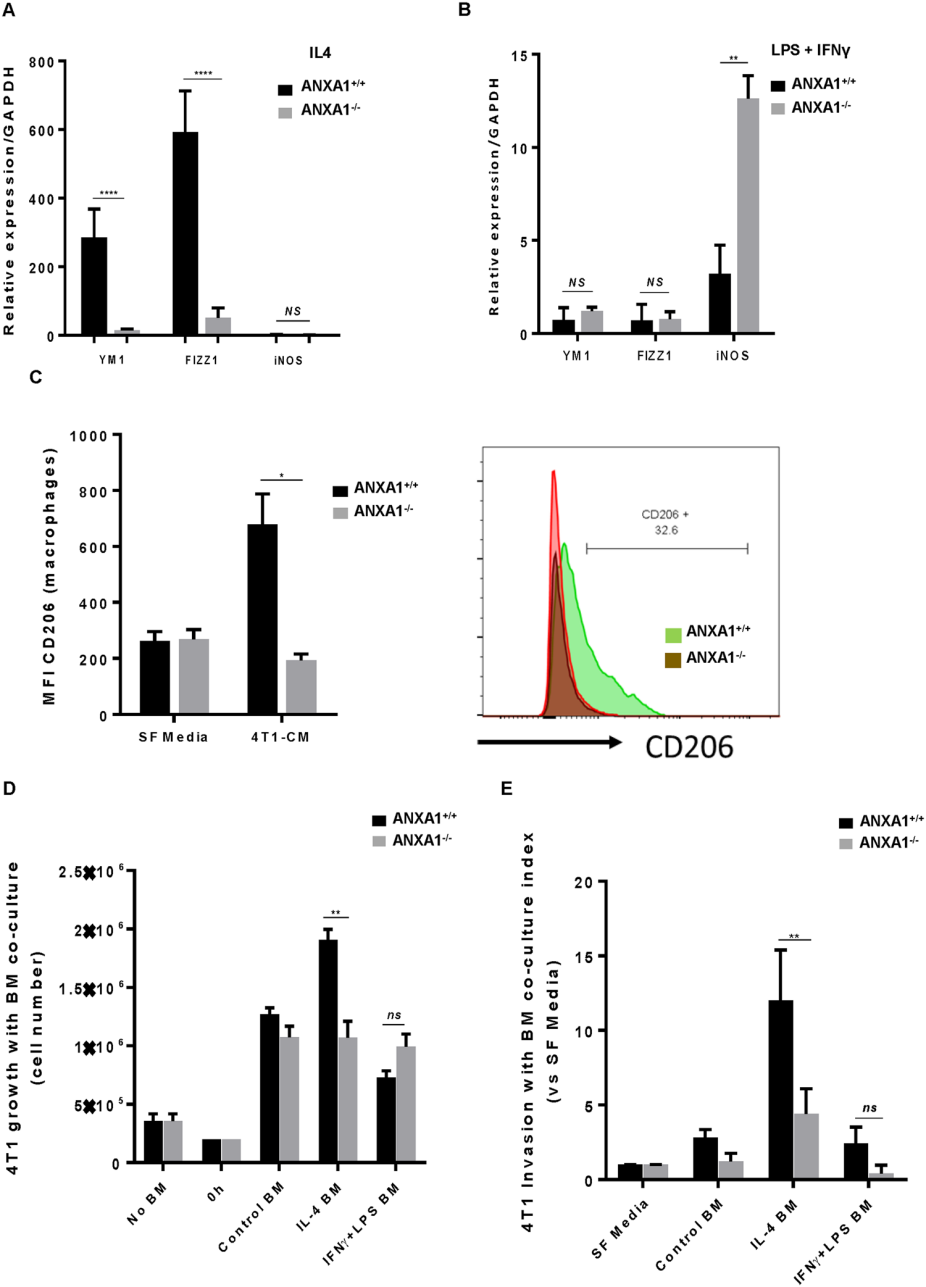
ANXA1 enhances M2 polarization and macrophage-stimulated breast cancer cell proliferation and invasion. BMDM were isolated from WT and ANXA1^−/−^ mice and treated with (**A**) IL4 (20 ng mL^−1^) or (**B**) LPS (10 ng mL^−1^) + IFNγ (50 ng mL^−1^) for 24 h. Phenotype for distinct macrophages were determined by mRNA expression of the primary classically M1 (iNOS) and activated M2 (YM1 and FIZZ1) markers. Results are expressed in mRNA levels after normalization with GAPDH by RT-qPCR (ΔΔCt method). (**C**) CD206+ cell expression was measured by flow cytometry upon 4T1-conditioned media treatment in BMDM isolated from WT and ANXA1^−/−^ mice. BMDM isolated from WT and ANXA1^−/−^ mice were treated with IL4 (20 ng mL^−1^) or LPS (10 ng mL^−1^) + IFNγ (50 ng mL^−1^) prior to transwell assay. 4T1 cells was either seeded at the bottom to allow for growth (**D**) or at the top to assess invasion (**E**). Data shown are mean ± SD (N = 4). *P ≤ 0.05, **P ≤ 0.01, ANOVA.

Annexin-a1 is a multifunctional molecule involved in a range of cellular signal transduction pathways, particularly in inflammation, innate and adaptive immune system, tumour progression and metastasis^32^. Previous studies shown that expression of ANXA1 is increased in certain cancers such as pancreatic and gastrointestinal cancer^33,34^, and decreased in others such as esophageal and prostate cancer^35,36^. To establish whether ANXA1 is required for macrophage polarization, the ability of lipopolysaccharide (LPS), gamma interferon (IFN-γ) to induce M1 polarization and interleukin 4 (IL-4) to induce M2 polarization was examined in murine bone-marrow-derived macrophages (BMDM) from WT BALB/c mice (ANXA1^+/+^) or mice deficient in ANXA1 (ANXA1^−/−^). IL4 treatment upregulated M2 markers YM1 and Retnia (Fizz1) in BMDM from ANXA1^+/+^, whereas macrophages derived from ANXA1^−/−^ mice were less sensitive to IL4-induced M2 polarization. Low iNOS levels were observed upon IL4 treatment in both BMDM from ANXA1^+/+^ and ANXA1^−/−^ mice (Fig. 3A), and LPS + IFNγ stimulation enhanced iNOS expression more significantly in ANXA1^−/−^ compared to ANXA1^+/+^ (p < 0.01, Fig. 3B), suggesting that in the absence of ANXA1, a polarization shift to M1-phenotype, rather than M2 phenotype is observed.

To investigate whether the effect of 4T1-CM on macrophage polarization is ANXA1-dependent, BMDM from ANXA1^+/+^ and ANXA1^−/−^ mice were treated with 4T1-CM for 24 h and CD206 expression (M2 marker) was analysed by flow cytometry. CD206 expression were significantly increased 24 h post-treatment with 4T1-CM in ANXA1^+/+^ cells, but not in macrophages from ANXA1^−/−^ mice (Fig. 3C). These data indicate that ANXA1 is required for the macrophage phenotype shift from M1 to M2 by 4T1-CM.

We further determined whether ANXA1 N-terminal peptide product Ac2-26 could induce M2 macrophage polarization. Macrophages were treated with ANXA1 peptide Ac2-26 (1 µM) and mRNA levels of IL-12 and Arg-1 was evaluated by RT-qPCR. At 24 h post-treatment, the overall polarization of macrophages shifted to M2, as they expressed high levels of Arg1 and low levels of IL-12 mRNA (Supplemental Fig. 2A,B). These data implies that ANXA1 itself can induce M2 phenotypic shift.

### ANXA1 is required for functional M2 macrophage dependent promotion of tumour growth and invasion

To address if macrophages can influence tumour cell proliferation and migration *in vitro*, and if this is dependent on ANXA1, macrophages derived from ANXA1^+/+^ and ANXA1^−/−^ mice were co-cultured in a transwell separation experiment with 4T1 breast cancer cells. BMDM were polarized for 24 h to M1 or M2-phenotype upon treatment with IFN-γ + LPS or IL4, respectively. 4T1 cells co-cultured with ANXA1^+/+^ M2-polarized macrophages exhibited enhanced growth and invasion compared to 4T1 cells treated with non-polarized media. However, 4T1 cells co-cultured with ANXA1^−/−^ M2-polarized macrophages demonstrated significantly lower growth and invasion than ANXA1^+/+^ macrophages (Fig. 3D,E). Taken together, these data suggest that macrophage ANXA1 plays an important role in the tumour microenvironment which can promote breast cancer growth and migration, possibly through the induction of macrophage polarization to an alternatively activated immunosuppressive phenotype (M2).

### CCL5 induces ANXA1 expression and modulates macrophage polarization

**Figure 4:**
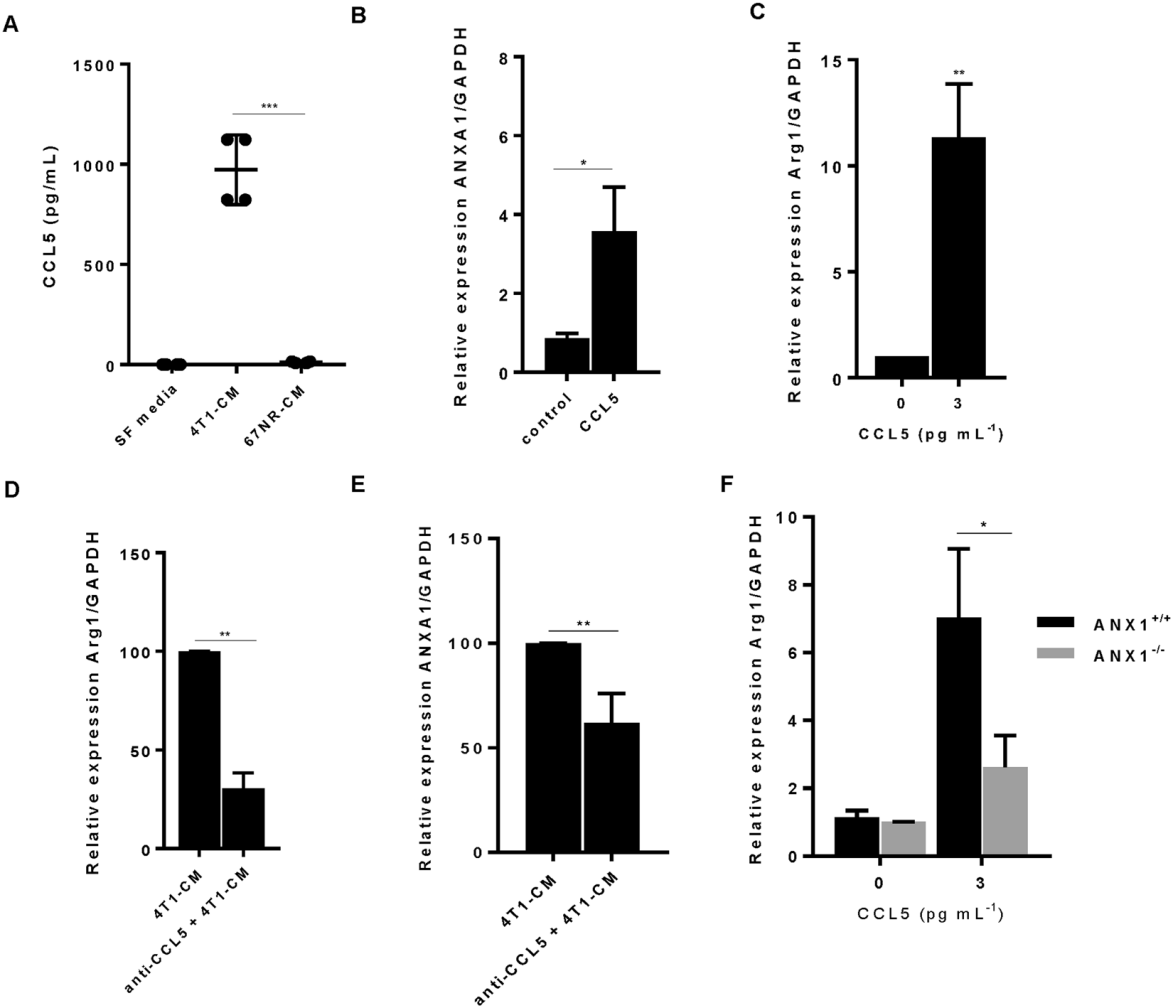
CCL5 is secreted from tumours and induces alternative macrophage polarization. (**A**) CCL5 in 4T1-metastatic and 67-NR-nonmetastatic breast cancer cell culture supernatant was quantified by ELISA. Raw 264.7 macrophages were treated with recombinant protein CCL5 (3 pg mL^−1^) for 24 h and (**B**) ANXA1 or (**C**) Arg1 expression was quantified by RT-qPCR. Macrophages were treated with 4T1-conditioned media for 24 h in the presence and absence of CCL5-blocking antibody (1 µg mL^−1^) and (**D**) Arg1 or (**E**) ANXA1 expression was quantified by RT-qPCR. BMDM were isolated from WT and ANXA1^−/−^ mice and treated with recombinant protein CCL5 (3 pg mL^−1^) for 24 h. Arg1 expression was quantified by RT-qPCR (**F**). Data shown are mean ± SD (N = 4). *P ≤ 0.05, **P ≤ 0.01, ANOVA.

The pro-inflammatory cytokine profile of the tumour microenvironment is extremely important to translate signals that communicate with immune cells, leading to inflammation and cancer progression. We therefore, examined the cytokine profile of conditioned media derived from 4T1 cells. Using a cytokine array, we observed that 4T1-CM contained 3-fold higher levels of CCL5 compared with serum-free media (Supplemental Fig. 3). In line with data obtained from the cytokine array, our validated ELISA results demonstrate that CCL5 was highly secreted by 4T1 cells (Fig. 4A).

It has been reported that CCL5 expression is observed in tissues and plasma of patients with advanced stage of disease^30^, similar to ANXA1 in breast cancer. We next sought to investigate whether CCL5 could regulate ANXA1 expression and macrophage polarization in response to 4T1-CM. ANXA1 mRNA expression in macrophages was upregulated upon recombinant CCL5 treatment (10 pg ml^−1^) (Fig. 4B). The levels of CCL5 from ANXA1^−/−^ BMDM were similar to those from ANXA1^+/+^ BMDM, indicating that ANXA1 does not regulate CCL5 expression (Supplemental Fig. 4). Recombinant CCL5 upregulated M2-marker (Arginase 1) in macrophages (Fig. 4C), and the expression of Arg1 and ANXA1 upon 4T1-CM treatment was prevented by a CCL5 neutralizing antibody (anti-CCL5) (Fig. 4D,E). To determine if CCL5 effect on M2-marker (Arginase 1) is ANXA1-dependent, Arg1 mRNA expression was measured upon recombinant CCL5 treatment in ANXA1^+/+^ and ANXA1^−/−^ BMDM. Arg1 mRNA expression levels from ANXA1^+/+^ BMDM were upregulated upon CCL5 treatment, whereas ANXA1^−/−^ BMDM were unresponsive to CCL5 recombinant protein (Fig. 4F). Taken together, these data indicate that CCL5 is able to a crosstalk between CCL5 and ANXA1 to promote macrophage polarization.

### ANXA1 and 4T1-CM enhances ERK and NF-κB activation via FPR2

**Figure 5:**
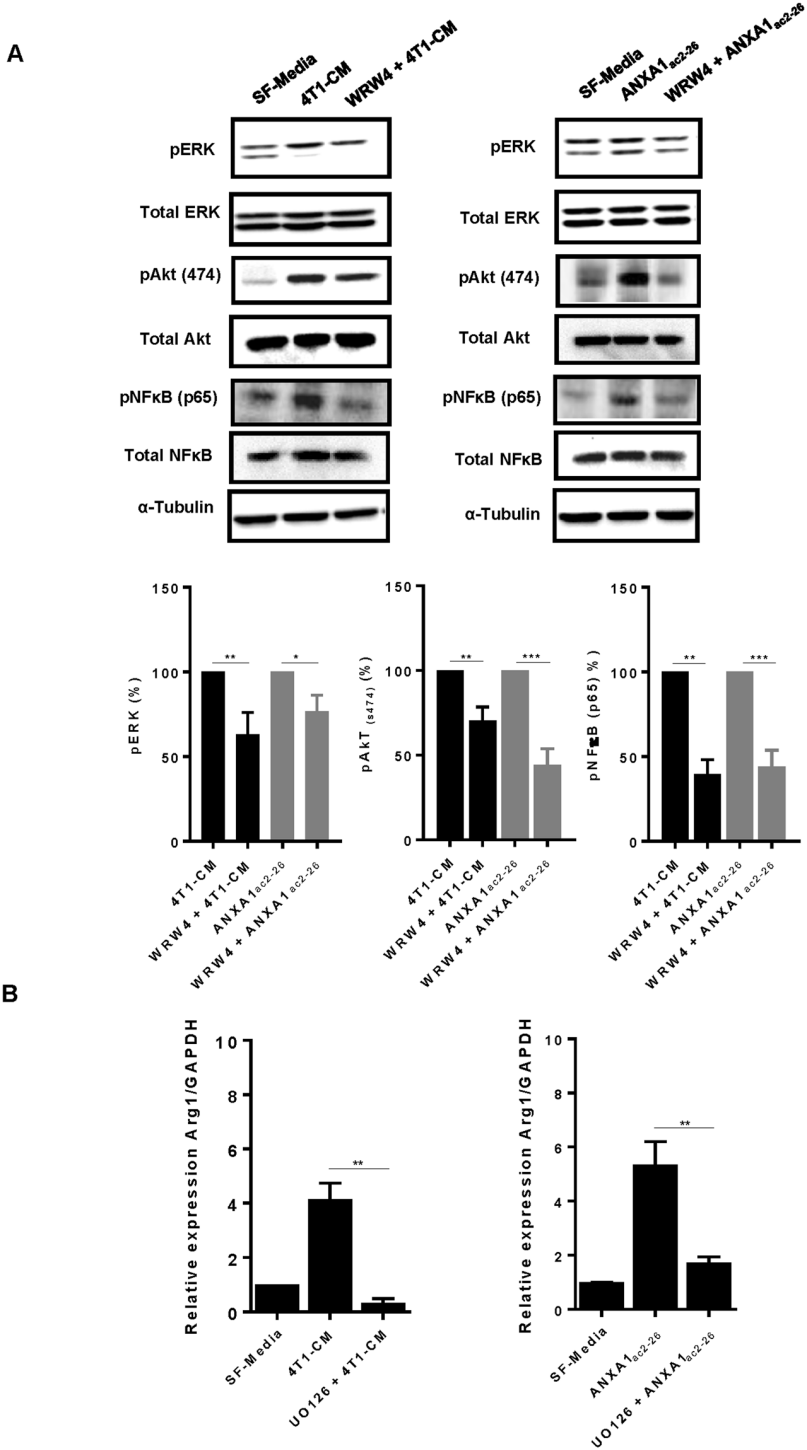
FPR2 and ERK activation is required for macrophage polarization. (**A**) RAW 264.7 macrophages were treated with 4T1-conditioned media or ANXA1 peptide ac2-26 (1 µg mL^−1^) in the absence or presence of WRW4 (10 µg mL^−1^). Cell lysates were subjected to immunoblot analysis using phosphospecific antibodies for ERK, AKt (473) and NFκB (p65). Blots and densitometry analysis are representative of 4 different experiments. (**B**) RAW 264.7 were pre-treated with ERK inhibitor, UO126 (5 µM), before treatment with 4T1-conditioned media or ANXA1 peptide ac2-26 (1 µg mL^−1^) for 24 h. M2 marker (Arg1) was quantified by RT-qPCR (ΔΔCt method). Data shown are mean ± SD (N = 4). **P ≤ 0.01, ANOVA.

We next explored potential signalling pathways to identify the mechanism by which ANXA1 enhances tumour cell migration. Naïve Raw 264.7 macrophages were treated with 4T1-CM or ANXA1 peptide ac2-26 for 24 h and phosphorylation of extracellular signal-regulated kinase 1/2 (ERK1/2), phosphoinositide 3-kinase (PI3K) and nuclear factor- κ beta (NF-κB) was explored. As shown in Fig. 5A, 4T1-CM or Ac2-26 peptide markedly induced phosphorylation of ERK1/2, Akt and NF-κB (p65), which was inhibited by FPR2 antagonist WRW4. Consistent with this, macrophages treated with 4T1-CM or ANXA-1 Ac2-26 exhibited marked upregulation of Arg1 mRNA expression (M2 marker), which was abrogated with pre-treatment of ERK1/2 inhibitor UO126 (5 µM) (Fig. 5B). These data indicate that FPR2-ERK signalling axis is involved downstream of ANXA1, which may be important in macrophage polarization.

### ANXA1 enhances breast tumour growth and inhibits macrophage activation *in vivo*

**Figure 6:**
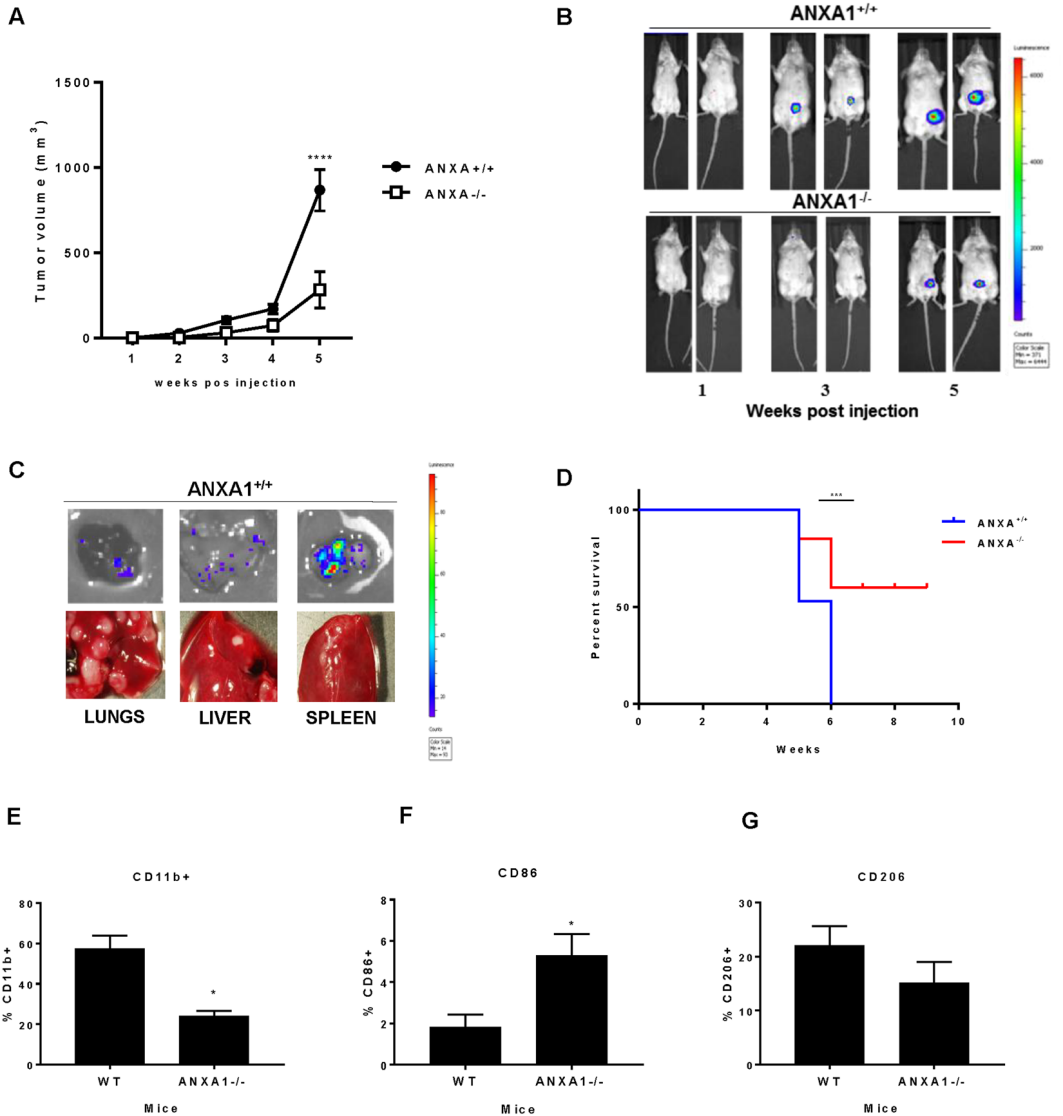
ANXA1 regulates syngeneic 4T1 tumour growth and metastasis *in vivo*. (**A**) BALBc WT and ANXA1^−/−^ mice were injected with 4T1 murine breast cancer cells containing a luciferase promoter into the mammary fat pad. Tumour volumes were calculated by LxWxW/2. (**B**) Bioluminescence imaging for mice from control and ANXA1^−/−^ group at week 1, 3 and 5 post-injection. (**C**) Representative bioluminescence image and pictures from lungs, liver and spleen after week 6 post-injection. (**D**) Survival curves were plotted for both groups (n = 6–8 per group, repeated twice). (**E**) Analysis of the percentage of CD45+ cells which are CD11b+. The percentage of (**F**) CD86+ (M1) or (**G**) CD206+ (M2) macrophages obtained from CD11b+ CD45+ population. Data shown are mean ± SD (n = 4). ****P ≤ 0.001, ANOVA.

Finally, to analyse the potential impact of tumor microenvironment derived ANXA1 on the regulation of tumour growth *in vivo*, 4T1 murine breast cancer cells which harbor a luciferase promoter (4T1-12B) were injected into the 4^th^ left mammary gland of female BALB/c WT and ANXA1^−/−^ mice. After injection, tumour growth was measured by manual measuring and bioluminescence imaging every week for 6 weeks. At 5 weeks post injection, tumour growth was significantly higher in the ANXA1^+/+^ group compared to ANX1^−/−^ group (Fig. 6A). Bioluminescence imaging confirmed that the ANXA1^+/+^ mice injected with 4T1 murine breast cancer cells exhibited increased luminescence compared with ANXA1^−/−^ mice (Fig. 6B). Similarly, metastasis in isolated lungs, liver and spleen were observed in ANXA^+/+^ mice after 6 weeks (Fig. 6C), whereas metastasis was not observed in ANXA1^−/−^ mice (data not shown). Consistent with this, ANXA1^−/−^ mice survived longer than ANXA1^+/+^ mice post-injection (Fig. 6D). To establish a direct connection between tumour growth and macrophage polarization, macrophages were isolated after 5 weeks post injection and the immune signature of the tumours were determined. Phenotype analysis of the tumour showed a smaller CD11b+ population derived from the tumour from ANXA1^−/−^ mice (Fig. 6E), and in this CD11b+ population, CD206+ expression levels were similar while CD86+ expression was significantly higher in the ANXA1^−/−^ mice group (Fig. 6F,G), suggesting that in the absence of ANXA1, the polarization of macrophages in the tumour are more skewed to M1-phenotype. This data strongly indicates that ANXA1 in the tumour microenvironment plays an important role in the development, progression and metastasis of breast cancer.

## Discussion

A hallmark of TAM-associated inflammation is the infiltration of leukocytes and stromal cells, which results in the enhanced macrophage recruitment and tumour development^11^. TAM infiltration are correlated with poor patient prognosis and metastasis in breast carcinoma^14^, and these effects may be due the ability of TAM to secrete cytokines, growth factors, chemokines and proteins that can stimulate cancer proliferation and cell invasion. The plasticity of macrophages polarization is rapid and occur at the levels of gene expression, protein, metabolite and microbicidal activity in response to changes in the cytokine environment^1–7,10,11^. The molecular mechanisms underlying the contribution of an inflammatory tumour microenvironment is not totally elucidated. Therefore, the characterisation of the phenotype of TAM is essential to the understanding of tumour-derived signals polarization of innate and adaptive immunity in tumour progression.

ANXA1 plays an important role in tumour development and progression of basal-like breast cancer^37^. We have shown previously that suppression of ANXA1 in highly metastatic breast cancer cells impedes migration and metastasis capabilities *in vitro* and *in vivo* ^21^. In the current study, we evaluated the effects of ANXA1 signalling on macrophage polarization in the tumour microenvironment. Our data demonstrates that ANXA1 is able to polarize macrophages to an alternatively activated subtype (M2), which binds to the G protein-coupled receptor, FPR2, mediating cellular effects in paracrine manner. Furthermore, our *in vivo* experiments confirm the ability of tumor microenvironment or host ANXA1 to control tumour growth and metastasis.

CCL5/RANTES is part of the CC chemokine family protein that interacts with G protein-coupled receptors CCR1, CCR3 and CCR5 in many cell types^38,39^. CCL5 has been proposed to be a novel and promising therapeutic target for breast cancer^39,40^. The implications of the chemokine CCL5 promotes progression and metastasis is a subject of debate. A previous study suggested that host-derived CCL5 promotes breast cancer growth and metastasis by restraining the normal differentiation of myeloid-derived suppressor cells subsets in a 4T1 mammary carcinoma model that mimics the triple-negative breast cancer in patients^39^. As CCL5 is able to mediate cross-talk between the tumour cells and the tumour microenvironment^40^, it may be possible that CCL5 modulates macrophage polarization in our study, as we have shown that CCL5 is the major chemokine secreted by 4T1 carcinoma cells and is able to induce macrophage polarization to an alternatively activated subtype. This was prevented by CCL5 blockade which led to the skewing of TAM phenotype towards the classically activated or M1 phenotype. Furthermore, macrophages treated with recombinant CCL5 exhibited increased ANXA1 expression, suggesting a crosstalk between CCL5 and ANXA1. Recently, in a very elegant study, a novel pro-resolving mechanism revealed that ANXA1 plays a new role as a potent monocyte chemoattractant to orchestrate the resolving phase of acute inflammation^41^. Consistent with this, our study reveals a new dimension between ANXA1 and CCL5 to modulate macrophage polarization and tumour cell proliferation. Whether the effects of ANXA1 on macrophage polarization are related to their shared abilities with chemokines (CCL5) or other target molecules remains to be established.

Due to their tissue expression in invasive breast cancer, ANXA1 and FPR2 have become a target for drug discovery. We have shown previously that ANXA1 induces the constitutive activation of NF-κB and subsequent effects on migration and metastasis in breast cancer^21^. In addition, ANXA1 can enhance ERK and RhoA activity in breast cancer cells^42^. Previous studies in different cell types indicate that activation of FPR2 may stimulate a number of signalling pathways, including ERK1/2, PI3K and MAPK signalling^28,43^. Indeed, it has been reported that ANXA1 associated with NF-κB FPR2 activation by distinct ligands can trigger the GPCR-mediated signalling cascade to modulate cytokine signalling and tumour microenvironment, resulting in macrophage polarization and tumorigenesis^8^.

In recent years debate has surround between ANXA1 expression and function in cancer. Recent studies provide evidence linking ANXA1 as an endogenous inhibitor of NF-κB that can be induced by glucocorticoids and modified nonsteroidal anti-inflammatory drugs (NSAIDs) in colon and prostate cancer cells^44^. Our study demonstrates that FPR2 activation by 4T1-CM or ANXA1 peptide Ac2-26 activates ERK1/2-Akt- NF-κB, which in turn may facilitate macrophage polarization into an M2 subtype. Whether the effect of ANXA1 on NF-κB or other signalling molecules are related to cancer tissue specificity remains to be established. It is important to note that ANXA1 is highly expressed in metastatic and triple negative breast cancer cells^21^, where their migration is dependent on TAM phenotype and function. It is tempting to speculate that the analysis of a classically to alternatively activated TAMs spectrum is likely further controlled by temporal and spatial variables within the microenvironment. Therefore, tumours of different origin are heterogeneous in their interaction with the host based on their ability to release factors, such cytokines, into macro and micro-environment to elicit specific host responses, which may be critical for the tumour-promoting activity. Our study provides evidence that ANXA1 modulates TAM function and phenotype through FPR2-ERK signalling, involving CCL5 in the microenvironment, suggesting a potential new axis for targeting cancer therapy.

## Methods

### Animals

All animal work was approved by the Institutional Animal Care and Use Committee and followed National Advisory Committee for Laboratory Animals Research (NACLAR) approved Institutional Animal Care and Use Committee (IACUC) protocols at the National University of Singapore. All experiments were performed in accordance with relevant guidelines and regulations. BALB/c mice (8-12 weeks) were obtained from Laboratory Animal Centre (Singapore). ANXA1^−/−^ mice on BALB/c background (backcrossed over 10 generations) were a gift from Prof. Roderick Flower, QMUL, UK. MMTV-Wnt mice were obtained from Prof David Virshup Laboratory (Duke NUS, Singapore) and were maintained by backcrossing to C57BL/6. Mice were kept on a 12-h light/dark cycle with food and water provided ad libitum and maintained under pathogen-free conditions in the animal housing unit.

### Cell Culture

Murine cell lines 4T1 and RAW 264.7 were obtained from American Type Culture Collection (ATCC, Manassas, VA, USA). Sublines from the same mammary tumour as 4T1, 67NR was a kind gift from Prof Jeal Paul Thiery, IMCB, A*STAR, Singapore. 4T1-12B cells stably transfected with a luciferase plasmid was a kind gift from Dr Gary Sahagian from Tufts University, USA. RAW 264.7 cells were grown as monolayers in Dulbecco’s Modified Eagle’s Medium (DMEM, Serana, Australia), while 4T1 and 67NR were cultured in Roswell Park Memorial Institute (RPMI, Serana, Australia) medium supplemented with 10% heat-inactivated fetal bovine serum (FBS, Biowest LLC, Kansas, MO, USA), 1% penicillin-streptomycin (GE Healthcare Life Sciences, HyClone Laboratories, Utah, USA) at 37 °C in a humid atmosphere containing 5% CO2. The cell lines were regularly authenticated through cell morphology monitoring, growth curve analysis and species verification. ANXA1 peptide ac2-26 and FPR2 antagonist WRW4 were obtained from Tocris Bioscences (Bristol, UK).

### Isolation of Bone Marrow-derived macrophages (BMDM)

BMDMs from wild-type and ANXA1^−/−^ mice (8–12 weeks) were obtained by flushing the femurs and tibias of mice with DMEM media, as previously described^45^. Briefly, red blood cells were removed through osmotic lysis and the bone marrow cell suspension was washed twice with PBS, and cultured with BMDM media (DMEM medium containing 10% FBS (v/v), 100 U/mL penicillin, 100 µg mL^−1^ streptomycin, 2 mM L-glutamine and 20% L-929-conditioned DMEM (v/v) as a source of M-CSF). After 3 days of culture, the cells were supplemented with fresh BMDM media. At day 7, culture media and non-adherent cells were removed and the remaining adherent cells were replenished with fresh BMDM media before experiments.

### Generation of tumour-conditioned media

4T1 and 67NR murine carcinoma cells were cultured in RPMI media containing 10% FBS and 1% penicillin-streptomycin. After 3 days of culture and between 80–90% confluence, the media was removed and replaced with fresh media containing 1% FBS and 1% penicillin-streptomycin. After 24 h, the conditioned-media (CM) was collected and passed through a filtropure syringe filter membrane (0.2 µm) and stored at −80 °C prior experiments.

### RNA extraction and RT-qPCR

Total RNA was extracted from the cells by using RNeasy kit (Qiagen, Limburg, Netherlands) according to the manufacturer’s protocol. The quantitative and qualitative RNA analyses were performed by using NanoDrop 1000 spectrophotometer (Thermo Fisher Scientific, Massachusetts, USA). Total RNA (1 µg) was used to synthesize cDNA by using a reverse transcription kit (GoTaq® qPCR master mix, Promega Corporation, Madison, USA) as previously described^22^. The results were normalized to the expression of glyceraldehyde-3phosphate dehydrogenase (GAPDH). The amplification program was as follows: 95 °C for 5 min followed by 40 cycles of 95 °C for 10 s, 60 °C for 10 s and 72 °C for 10 s. The specificity of the assay was confirmed by melting curve analysis at the end of the amplification program. Primers for ANXA1, PPARγ, INOS, Arg1, IL12, Fizz, YM1 are described in supplemental file (Table S1).

### Cytokine detection

4T1 and 67NR-conditioned media were analyzed for cytokine CCL5 using mouse CCL5 Enzyme-linked immunosorbent assay (ELISA) standard system (Peprotec, NJ, USA), following the manufacturer’s instructions.

### Western Blot

Cells were washed twice with ice cold 1 × phosphate buffer saline (PBS). Proteins were extracted from the cells by using RIPA lysis and extraction buffer as previous described^22^. Protein concentration was estimated according to the Bradford’s protein assay (BioRad Laboratories, Hercules, California, USA). Equal amounts of protein from each sample were subjected to 10% SDS-PAGE at a constant voltage of 125 V. The proteins were transferred onto nitrocellulose membranes (Bio-Rad, Hercules, CA, USA). Proteins were determined by Western blotting with specific antibodies, and expression signals were obtained by enhanced chemiluminescence. Protein expression was normalized to α-tubulin levels. Specific antibodies against phosphor-Akt-Ser 473, Akt, phosphor-ERK, ERK, phosphor-NFΚB p-65 (Cell signalling Technology), iNOS (Invitrogen), PPARγ (Santa Cruz), α-tubulin (Abcam) and Arg1 (Sigma) were used for immunoblot analysis.

### Transwell co-culture assay

*In vitro* cell migration assay was performed using the Transwell system (24-wells, 8-μm pore size with polycarbonate membrane; Corning Costar, Lowell, MA, USA). Briefly, BMDM and 4T1 cells were harvested and suspended in serum-free media and 1 × 105 cells were added to the upper wells. The 4T1 and BMDM-conditioned media were pre-treated with IFNγ (Peprotech) + LPS (Sigma) or IL4 (R&D systems) and mixed with media (v/v 1:1), prior addition to the lower chamber. After 24 h, the cells attached to the lower surface were counted. The number of cells migrated were acquired in five randomized fields using an Olympus light microscope13 to obtain the invasion index.

### Isolation of tumour-associated macrophages (TAM)

Tumors were mechanically dissociated and strained through a 40 µm nylon mesh before digestion into single cells with collagenase type II (0.5 mg mL^−1^), collagenase type IV (0.5 mg mL^−1^), hyaluronidase (10 U/mL) and DNase I (0.01 mg mL^−1^) for 2 h at 37 °C. The dissociated cells were collected, lysed by RBC lysis buffer. Phenotypic analyses were carried out on isolated macrophages for distinct M1/M2 populations as indicated. Cells were stained in ice-cold PBS containing FCS (2%) and EDTA (2 mM) using appropriate antibody-fluorophore conjugates. Multiparameter analysis was performed in a Fortessa analyser (BD Biosciences) and analysed with FlowJo software (Tree Star, Ashland, USA). The following antibodies were purchased from Biolegend (San Diego, USA): anti-CD45 (clone 30-F11), anti-GR-1 (clone RB6-8C5), anti-F480 (clone BM8) anti-CD206 (clone C068C2) and anti-CD86 (clone GL-1) while anti-CD11c (clone N418) and Ly6G (clone Ia8) were purchased from ebioscience (ThermoFisher Scientific, Waltham, MA, USA).

### *In vivo* bioluminescence imaging of 4T1 tumour

Sixteen mice were randomized into two groups (WT and ANXA1 deficient mice, 8 mice per group). The mice were subcutaneously injected with stably transfected 4T1-luciferase cells (7500 cells per mouse) into the mammary fat pad and mice were monitored for up to 40 days. The size of the tumours and tissue metastasis were measured by a bioluminescence imaging assay using the Xenogen IVIS Spectrum Iamging System (Caliper Life Sciences) and manual measurement by Vernier caliper. Tumour volume was calculated as (length × width × width/2). Mice were killed by anaesthetic overdose either at the end of the study or earlier if they displayed significant weight loss, signs of distress or palpable tumours ≥1.5cm in diameter.

### Statistical analysis

Results are the means ± SEM of three independent experiments performed in triplicate. Statistical comparisons between groups were made by using one-way ANOVA and Bonferroni post-tests were performed for intergroup significance. Unpaired two-tailed Student’s t-test wsa used for comparing two variables. The differences were considered statistically significant at *p < 0.05.

## Electronic supplementary material

Supplementary figures
