# Supplementary material for: Annexin-A1 enhances breast cancer growth and migration by promoting alternative macrophage polarization in the tumour microenvironment
Source: Sci Rep. 2017 Dec 20;7:17925. doi: 10.1038/s41598-017-17622-5 (PMC5738423; doi:10.1038/s41598-017-17622-5)
Supplement: Supplementary file 1 — Supplementary figures [file 41598_2017_17622_MOESM1_ESM.pdf]

Annexin-A1 enhances breast cancer growth and migration by promoting alternative macrophage polarization in the tumour microenvironment

Leonardo A. Moraes, Shreya Kar, Foo Sok Lin, Tong Gu, Yi Qian Toh, Patrick B Ampomah, Karishma Sachaphibulkij, Gracemary Yap, Olga Zharkova, Hakim M. Lukman, Anna-Marie Fairhurst, Alan Prem Kumar, Lina HK Lim

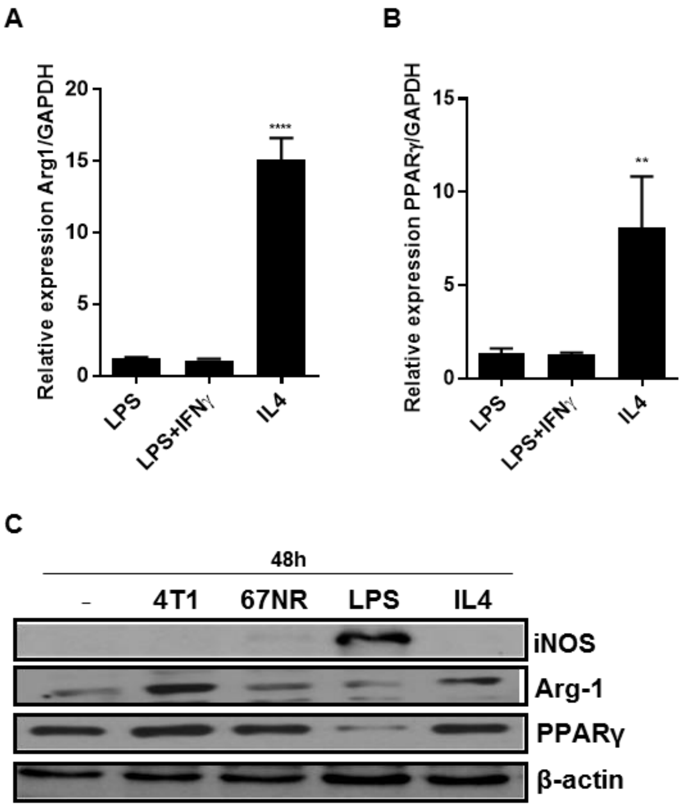

Figure 1 – 4T1 and 67NR-conditioned media effects on macrophage polarization. Phenotype for distinct macrophages were determined by mRNA expression of (A) Arg1 and (B) PPARγ after treatment with IL4 (20 ng ml^{-1}), LPS (10 ng ml^{-1}) or LPS (10 ng ml^{-1}) + IFNγ (10 ng ml^{-1}). Data represents relative gene expression after normalization with GAPDH by RT-qPCR (ΔΔCt method). (C) Protein expression of Arg1, PPARγ and iINOS were determined after treatment with 4T1 and 67NR-conditioned media, LPS (10 ng ml-1) or IL4 (20 ng ml-1) for 48h. Immunoblots are representatives of 3 experiments. Data shown are mean ±SD (n=4). **P≤0.01, ****P≤0.001, ANOVA.

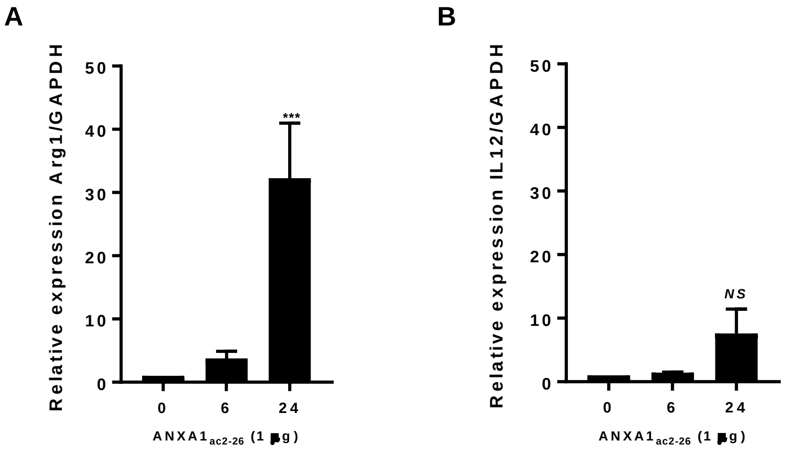

Figure 2 – ANX1 plays a role on macrophage polarization. RAW 264.7 macrophages were treated with ANXA1 peptide ac2-26 (1 µg mL-1) and phenotype for distinct macrophages were determined by mRNA expression of the primary classically M1 (IL12) and activated M2 (Arg1) markers. Results are expressed in mRNA levels after normalization with GAPDH by RT-qPCR (ΔΔCt method). Data shown are mean ± SD (n=4). ***P≤0.01, ANOVA.

Supplemental figure 3

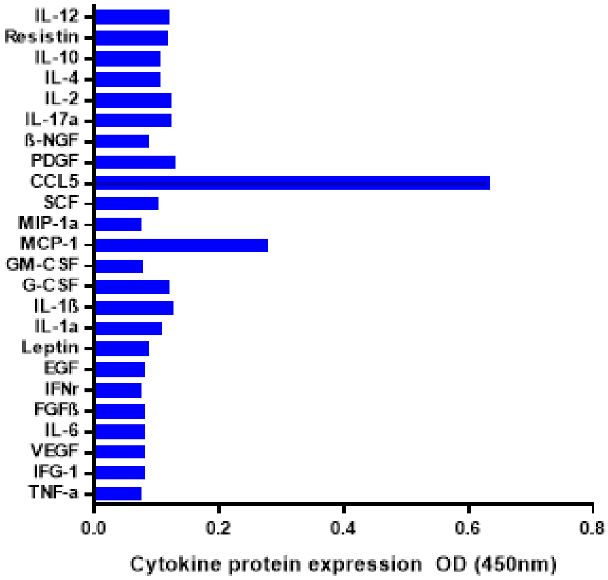

Figure 3 – Cytokine protein array derived from 4T1-breast carcinoma cells. 4T1 cells were cultured in RPMI for 48h and culture supernatants were collected and cytokine was measured by optical density transmission (OD).

Supplemental figure 4

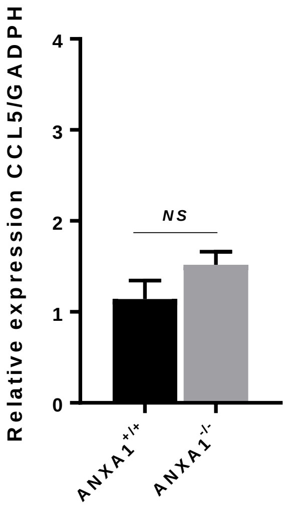

Figure 4 – Characterization of ANXA1-deficient macrophages. BMDM were isolated from WT and ANXA1$^{-/-}$ mice and chemokine CCL5 expression was quantified by RT-qPCR. Results are expressed in mRNA levels after normalization with GAPDH (A). Data shown are mean ±SD (n=3). *NS* (no significant, *t*-test.
